# Anti-malarial effect of semi-synthetic drug amitozyn

**DOI:** 10.1186/s12936-015-0952-4

**Published:** 2015-10-29

**Authors:** Sergey O. Tcherniuk, Olga Chesnokova, Irina V. Oleinikov, Anatoly I. Potopalsky, Andrew V. Oleinikov

**Affiliations:** Department of Biomedical Science, Charles E Schmidt College of Medicine, Florida Atlantic University, Boca Raton, FL USA; Department of Biological Sciences, Youth Academy of Sciences, Kiev, Ukraine; Institute of Molecular Biology and Genetics, National Academy of Sciences of Ukraine, Kiev, Ukraine; Institute of Health Promotion and Rebirth of People of Ukraine, Kiev, Ukraine

**Keywords:** Malaria, Amitozyn, Alkaloids, Microtubules, Isobolographic analysis

## Abstract

**Background:**

Malaria caused by *Plasmodium falciparum* is the most virulent form of malaria, leading to approximately a half million deaths per year. Chemotherapy continues to be a key approach in malaria prevention and treatment. Due to widespread parasite drug resistance, identification and development of new anti-malarial compounds remains an important task of malarial parasitology. The semi-synthetic drug amitozyn, obtained through alkylation of major celandine (*Chelidonium majus*) alkaloids with *N*,*N*′*N*′-triethylenethiophosphoramide (ThioTEPA), is a widely used Eastern European folk medicine for the treatment of various tumours. However, its anti-malarial effect has never been studied.

**Methods:**

The anti-malarial effects of amitozyn alone and in combination with chloroquine, pyrimethamine and artemisinin on the blood stages of *P. falciparum* were analysed. The cytostatic effects of amitozyn on parasites and various cancerous and non-cancerous human cells were compared and their toxic effects on unparasitized human red blood cells were analysed.

**Results:**

Obtained results demonstrate that amitozyn effectively inhibits the growth of blood-stage parasites with IC_50_ 9.6 ± 2, 11.3 ± 2.8 and 10.8 ± 1.8 μg/mL using CS2, 3G8 and NF54 parasite lines, respectively. The median IC_50_ for 14 tested human cell lines was 33–152 μg/mL. Treatment of uninfected red blood cells with a high dose of amitozyn (500 μg/mL) did not change cell morphology, demonstrating its non-toxicity for erythrocytes. The synergistic impact of the amitozyn/chloroquine combination was observed at growth inhibition levels of 10–80 %, while demonstrating a nearly additive effect at a growth inhibition level of 90 %. The combination of amitozyn with pyrimethamine has a synergistic effect at growth inhibition levels of 10–70 % and a nearly additive effect at a growth inhibition level of 90 %. The synergistic anti-malarial effect of the amitozyn/artemisinin combination was observed at growth inhibition levels of 10–40 % and a nearly additive effect at growth inhibition levels of 50–90 %.

**Conclusions:**

These in vitro results suggest that the semi-synthetic drug amitozyn, typically used for the treatment of tumours, is a potential anti-malarial candidate and warrants more detailed laboratory and pre-clinical investigations.

**Electronic supplementary material:**

The online version of this article (doi:10.1186/s12936-015-0952-4) contains supplementary material, which is available to authorized users.

## Background

In humans, malaria is caused by five species: *Plasmodium falciparum, Plasmodium malariae, Plasmodium ovale, Plasmodium vivax*, and *Plasmodium knowlesi* [1, 2]. Among infected individuals, *P. falciparum* is the most common species identified (about 75 %) followed by *P. vivax* (about 20 %) [3]. Moreover, *P. falciparum* represents the most virulent form of human malaria, causing the majority of deaths [4] although it has been shown recently that malaria induced by *P. vivax* may also be associated with potentially life-threatening conditions [5].

The first known anti-malarial treatment was infusion of bark obtained from plants of the *Cinchona* genus [6]. Its anti-malarial activity was attributed to the alkaloid quinine (QN), which was characterized in 1820 and used as the anti-malarial compound for extended periods of time. Later the QN was replaced by chloroquine (CQ)—a cheaper, synthetic analogue. Appearance of CQ-resistant strains led to the discovery of the new potent anti-malarial drug artemisinin (ART) in the 1970s. This natural sesquiterpene endoperoxide is currently one of the major anti-malarial drugs used around the world. However, *P. falciparum* resistance to ART and its derivatives has been described recently [7]. At present ART derivatives are commonly used in drug combinations with lumefantrine, amodiaquine, mefloquine, sulfadoxine-pyrimethamine and antibiotics to treat uncomplicated *P. falciparum* malaria and *P. vivax* in areas of CQ resistance [8, 9].

There is a large arsenal of synthetic and natural compounds possessing anti-malarial activity. These compounds belong to different chemical classes and have different mechanisms of action. It is already known that some anticancer drugs have strong anti-malarial effect. For example, antimetabolite methotrexate [10], paclitaxel [11], vinblastine [12], cisplatin [13], and bortezomib [14] demonstrate potent activity against the blood stages of *P. falciparum.*

In this work, the antiparasitic effect of the semi-synthetic drug amitozyn was studied. Obtained results demonstrate that amitozyn possesses anti-malarial activity and inhibits in vitro blood stage growth of *P. falciparum*. Earlier, it has been shown that amitozyn is an effective anticancer drug, which acts as a microtubule modulator [15]. In this work the microtubule depolymerization effect of amitozyn was observed at different blood stages of *P. falciparum,* which could be the principal cause of its anti-malarial effect. Combination of amitozyn with CQ showed a synergistic anti-malarial effect at growth inhibition levels of 10–80 % and a nearly additive effect at a growth inhibition level of 90 %. Amitozyn/pyrimethamine combinations provoked a synergistic anti-malarial effect at growth inhibition levels of 10–70 % and a nearly additive effect at growth inhibition levels of 80–90 %. Amitozyn/artemisinin combinations showed a synergistic anti-malarial effect at growth inhibition levels of 10–40 % and a nearly additive anti-malarial effect at growth inhibition levels of 50–90 %.

## Methods

### Materials

The semi-synthetic drug amitozyn was prepared as described previously at a concentration of 25 mg/mL [15]. CQ, pyrimethamine, ART, monoclonal mouse anti-α-tubuline (T9026), and polyclonal anti-γ-tubuline antibodies (T3559) were purchased from Sigma. Advanced RPMI Medium 1640, Alexa Fluor 488 goat anti-mouse and Alexa Fluor 594 goat anti-rabbit antibodies were from Invitrogen. 4′,6-Diamidino-2-phenylindole, dihydrochloride (DAPI) and LDH cytotoxicity kit were from Termo Scientific Pierce. EGM-2 medium was from Lonza. The polyclonal rabbit anti-human RBC antibodies were from Rockland. Human blood O^+^ and AB human serum were purchased from Valley Biomedical.

Human HeLa, KB3, HT29, HCT116, A549, IMR90, HUVEC, MESSA, and murine B16, GL26 and COS7 cell lines were purchased from ATCC. HCT116 p53(−/−) cells with homozygous knock-out of p53 were kindly provided by Dr D Skoufias (IBS, Grenoble, France). Taxol-resistant A549T12 cells were obtained with permission from Dr S Horwitz (Albert Einstein College of Medicine, New York, NY, USA). The MESSA Dx5 cells were kindly provided by Dr L Lafanechère (CNRS, UMR 5168/CEA/IRTSV, Grenoble, France).

### Ethics statement

Ethics approval was obtained from the Florida Atlantic University Institutional Review Board committee for using human erythrocytes for culturing malaria parasites. Blood was provided by Valley Biomedical.

### Parasite culture

CS2, NF54 and 3G8 (kindly provided by Dr J Smith) strains of *P. falciparum*, were grown in human O^+^ erythrocytes at 2 % haematocrit in complete medium containing RPMI 1640 supplemented with 10 % human serum and 40 μg/mL gentamicin sulphate as previously described [16]. Cultures were maintained at 37 °C in a gas mixture of 5 % CO_2_, 5 % O_2_ and 90 % N_2_. Synchronization was performed using 5 % d-sorbitol [16].

### In vitro drug susceptibility assays

The antiparasitic effects of amitozyn, CQ, pyrimethamine and ART were tested on the CS2, 3G8 and NF54 strains of *P. falciparum* by counting infected erythrocytes visualized by a standard Giemsa staining method [17]. The combinatory effect of amitozyn/chloroquine, amitozyn/pyrimethamine and amitozyn/artemisinin was analysed using the same method in the 3G8 strain. Non-synchronized parasites (0.5 %) with 2 % haematocrit were seeded in a 96-well plate and amitozyn (0–125 μg/mL), CQ (0–30 nM), pyrimethamine (0–20 nM), and ART (0–4 nM) were added to the media. Plates were incubated at 37 °C for 96 h with periodic change of media (containing appropriate drug concentration) every 24 h. DMSO (0.1 %) was used as a control. Parasitaemia was determined by light microscopy after Giemsa staining at time points of 24, 48, 72, and 96 h. Antiparasitic effect (APE) of drugs was measured in per cent and calculated by the equation APE (%) = 100 − RP, where RP is a per cent of parasitaemia relative to control (no drug).

### Cell viability and cytotoxicity assay

The antiproliferative effect of amitozyn was analysed in 14 different cell lines. HeLa, IMR90, GL26, COS7, and B16 cells were grown in DMEM medium supplemented with 2 mM l-glutamine, 1 % penicillin/streptomycin and 10 % FBS. KB cells were grown in the same medium supplemented with 20 % FBS. MESSA, MESSA (Dx5) MDR, A549, and A549T12 were cultivated in RPMI 1640 medium supplemented with 2 mM l-glutamine, 1 % penicillin/streptomycin and 10 % FBS. The medium for A549T12 cells was additionally supplemented with 2 nM paclitaxel. HCT 116, HCT 116 p53(−/−), and HT29 were cultivated in McCoy’s 5A medium supplemented with 1 % penicillin/streptomycin and 10 % FBS. HUVEC were grown in EGM-2 medium supplemented with 2 % FBS. All cell lines were maintained in a humid incubator at 37 °C in 5 % CO_2_.

To estimate the antiproliferative effect of amitozyn in human cells, the crystal violet cell viability assay was performed. Aliquots of 4–6 × 10^3^ cells/well were seeded in 96-well plates and incubated with 0–500 μg/mL of amitozyn, for 72 h. The cells were washed with PBS, fixed with formaldehyde (3.7 %) for 30 min, and stained with 0.1 % crystal violet for 10 min, washed abundantly with water, and dried at room temperature. Finally, a 10 % solution of acetic acid was added to each sample to dissolve the blue dye. The absorbance of the samples was measured spectrophotometrically at 595 nm for quantitative evaluation of cell viability. Cell viability was calculated by the following equation: Cell viability (%) = Q(Dr)/Q(ctrl) × 100, where Q(Dr) is quantity of viable cells after appropriate drug treatment, Q(ctrl) is quantity of viable cells in the control. To estimate the antiproliferative effect of amitozyn/chloroquine, amitozyn/pyrimethamine and amitozyn/artemisinin combinations in the human cells, the HUVEC culture was treated with amitozyn (0, 7.5, 15, 30, 60, 125, 250, 500 μg/mL) combined with above-mentioned drugs at molar ratio 1:1 for 72 h. Then the crystal violet cell viability assay was performed as described above. Growth inhibition (GI) was calculated by the following equation GI (%) = 100 − cell viability (%).

To analyse the cytotoxic effect of amitozyn on red blood cells (RBC), RBC (O^+^) at 2 % haematocrit were seeded in 96-well plates and incubated with 0, 30, 60, 125, 250, 500 μg/mL of amitozyn for 24, 48, 72, and 96 h. RBC incubated with complete RPMI supplemented with 0.1 % Triton X-100 were used as a positive cytotoxic control. At the desired time point the medium was removed and tested in triplicates using the lactate dehydrogenase (LDH) cytotoxicity kit. The results of cytotoxicity assay were calculated as described in the kit manual. The statistical significance of the difference between the control and treated groups was determined by Student t test. P value ≤0.05 was considered to be statistically significant.

### Isobolographic analysis

The combination index (CI) of amitozyn/chloroquine, amitozyn/pyrimethamine and amitozyn/artemisinin combinations was calculated at various levels of antiparasitic or growth inhibition effects (from 10 to 90 %) to evaluate their combination effects in the 3G8 strain of *P. falciparum* and in the HUVEC, respectively, as was described earlier [18]. CI was calculated by the equation CI_x_ = (D_A_/(D_x_)_A_) + (D_B_/(D_x_)_B_) + (D_A_ × D_B_)/((D_x_)_A_ × (D_x_)_B_) for a mutually non-exclusive interaction, where D_A_ is a dose of amitozyn used in combination with drug B (CQ, pyrimethamine or ART) required for the effect of X %, (D_x_)_A_ is a dose of amitozyn alone required for the effect of X %, D_B_ is a dose of drug B (CQ, pyrimethamine or ART) used in combination with amitozyn required for the effect of X % and (D_x_)_B_ is a dose of drug B (CQ, pyrimethamine or ART) alone required for the effect of X %. The synergistic, nearly additive or antagonistic effect was determined by the scale described earlier [19]. CI values from 0.9 up to 1.1 indicated nearly additive effect and CI values less than 0.9 indicated synergistic effects. All experiments were repeated at least three times.

### Immunofluorescence

To analyse the effect of amitozyn on the parasite microtubules, the RBC (2 % haematocrit) were infected with the 3G8 strain of *P. falciparum*. At 2 % parasitaemia the infected RBC were treated with amitozyn (0–250 μg/mL) for 24 h. Treated and untreated RBC were harvested and fixed by 4 % paraformaldehyde in PBS supplemented with 0.0075 % glutaraldehyde for 30 min at 37 °C [20]. After a few washes in PBS (1 mL), RBC were treated with anti-human RBC and anti α-tubulin antibodies (Abs) diluted 1:500 and 1:50, respectively, in the antibody buffer (PBS containing 3 % BSA, 0.05 % Tween and 0.02 % sodium azide) for 1 h at 37 °C.

Cells were subsequently stained with Alexa Fluor 488 and Alexa Fluor 594-conjugated goat anti-mouse and anti-rabbit secondary Abs at 1:500 dilution for 45 min at 37 °C. Finally, cells were counterstained with 1 μg/mL DAPI. Images were captured by LSM-510 META Zeiss confocal microscope and analysed using the ImageJ software.

## Results

### Amitozyn inhibits the growth of *Plasmodium falciparum* cultures in vitro

**Fig. 1:**
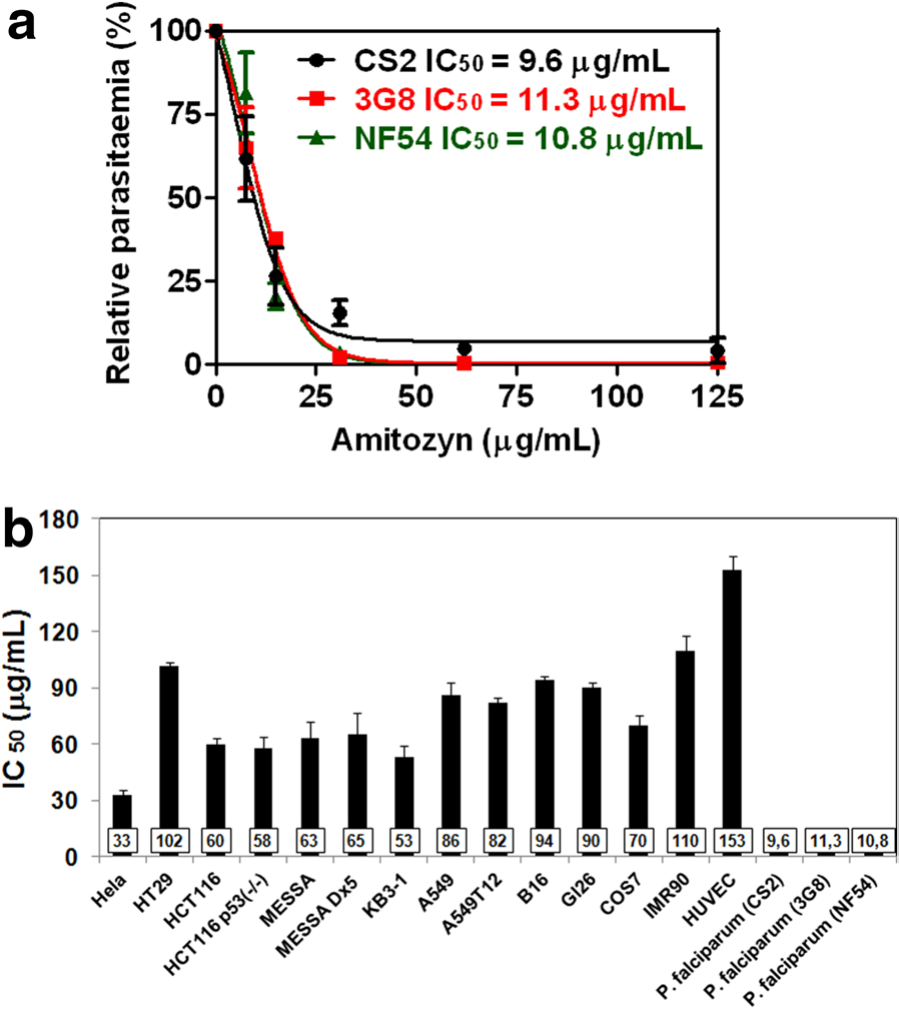
Antiproliferative effect of amitozyn on eukaryotic cells and culture of *Plasmodium falciparum.* **a** IC_50_ of amitozyn for CS2, 3G8 and NF 54 strains of *P. falciparum*. Human RBC at 2 % haematocrit were infected with CS2, 3G8 and NF54 strains of *P. falciparum.* At 0.5 % parasitaemia infected RBC were treated with 0–125 μg/mL amitozyn for 96 h and then stained by Giemsa’s method. Parasitaemia was analysed by inverted light microscope as described in “Methods”. The data were statistically treated and plotted on the graphs using GrahPad software. IC_50_ values were found from the graph. **b** Comparison of IC_50_ of amitozyn in eukaryotic cell cultures and in the culture of *P. falciparum* (CS2, 3G8 and NF54 lines). Eukaryotic cells were exposed to 0–500 μg/mL amitozyn. At 72 h cells were fixed with 3.7 % formaldehyde and stained with 0.1 % crystal violet. Ratio of viable cells was analysed as described in “Methods”. The value of IC_50_ was estimated from cell viability plots and presents the average value of the three independent experiments

The susceptibility assay in vitro was performed against *P. falciparum* (CS2, 3G8 and NF 54 parasite lines) in order to analyse the antiparasitic effect of amitozyn. Amitozyn treatment inhibited the proliferation of SC2, 3G8 and NF54 lines in a dose-dependent manner (Fig. 1a). Calculated IC_50_ for CS2, 3G8 and NF54 lines were 9.6 ± 2, 11.3 ± 2.8 and 10.8 ± 1.8 μg/mL respectively (Fig. 1a). Treatment with 30 μg/mL amitozyn for 96 h completely inhibited the growth of the three parasite lines used (Fig. 1a). Pretreatment of *P. falciparum* (3G8 strain) with 15 μg/mL amitozyn for 24 h inhibited the parasite growth even after abundant wash and release in drug-free medium for 144 h (Additional file 1). Pretreatment with the same concentration of amitozyn for 48 h drastically inhibited the *P. falciparum* growth in drug free medium but did not diminish the initial parasitaemia. Pretreatment of parasite culture with increased amitozyn concentration (30 μg/mL) for 24 h completely inhibited the parasite growth in the drug-free medium but similarly did not decrease the initial parasitaemia. However, pretreatment with amitozyn 30 μg/mL for 48 h diminished the initial parasitaemia during release in the drug-free medium for 144 h (Additional file 1). These results show that the type of antiparasitic effect (cytostatic or cytotoxic) of amitozyn depends on the drug concentration and length of treatment. It appears that amitozyn used in concentrations up to 15 μg/mL provoked a cytostatic effect, but at concentrations of 30 μg/mL amitozyn induced a cytostatic or cytotoxic effect depending on the length of treatment.

Importantly, all *P. falciparum* lines described above were significantly more susceptible to amitozyn than the wide spectrum of human and mouse cells (Fig. 1b). IC_50_ of amitozyn varied from 33 ± 3 μg/mL for HeLa cells up to 153 ± 8 μg/mL for HUVEC. These results suggest that amitozyn inhibits the proliferation of blood stage *P. falciparum* more effectively than proliferation of the human and animal cells.

### Amitozyn disrupts the microtubules of *Plasmodium falciparum*

**Fig. 2:**
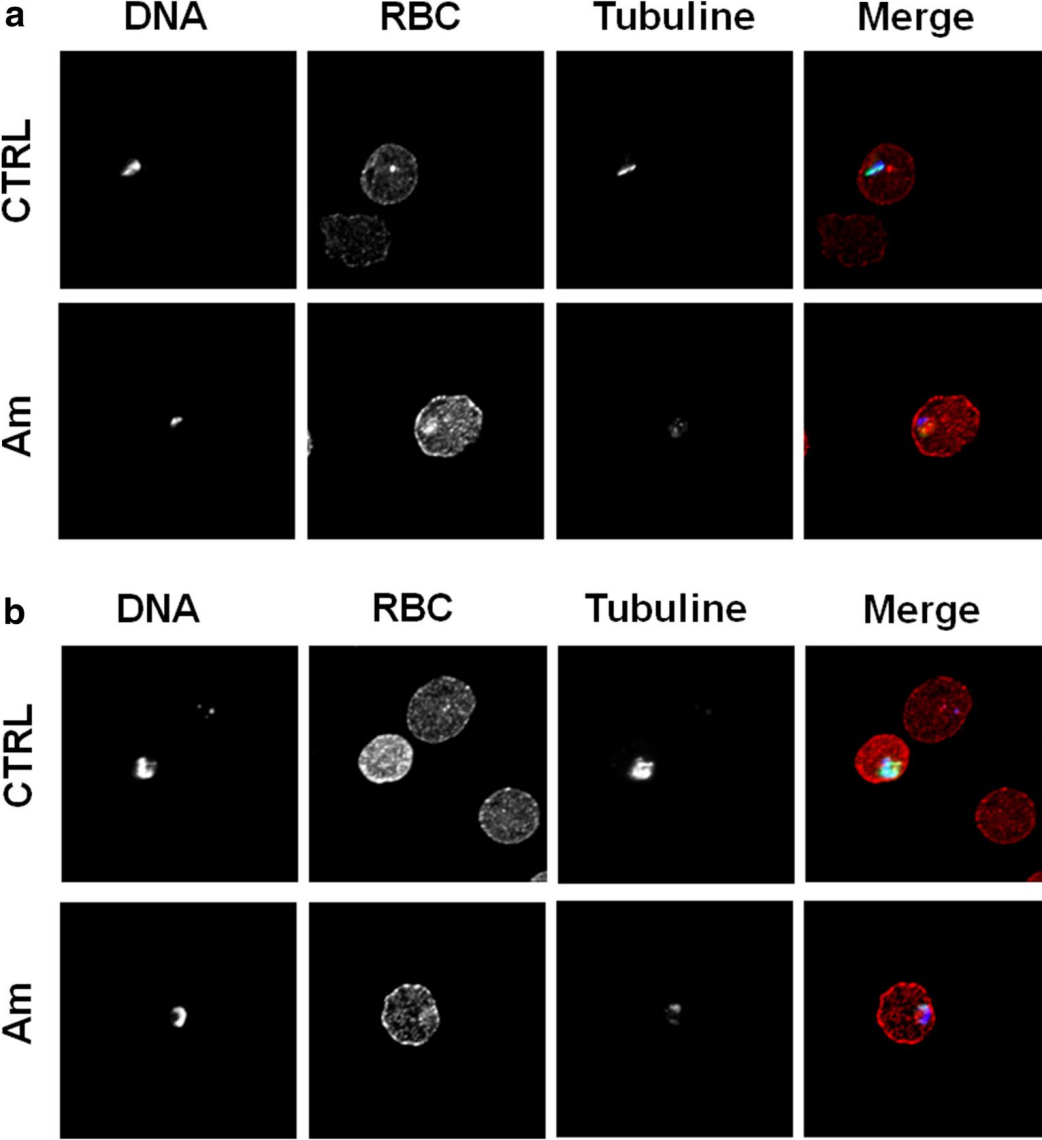
Effect of amitozyn on the microtubules of *Plasmodium falciparum.* Human RBC at 2 % haematocrit were infected with 3G8 strain of *P. falciparum.* At 0.5 % parasitaemia infected RBC (iRBC) were treated with 30 μg/mL amitozyn for 24 h. Untreated iRBC were used as a control. Fixed iRBC were stained with anti-α-tubulin (*green*), anti-RBC (*red*) and DAPI (*blue*) and analysed by confocal microscopy. **a** Rings and **b** trophozoite stages of *P. falciparum*, respectively

**Fig. 3:**
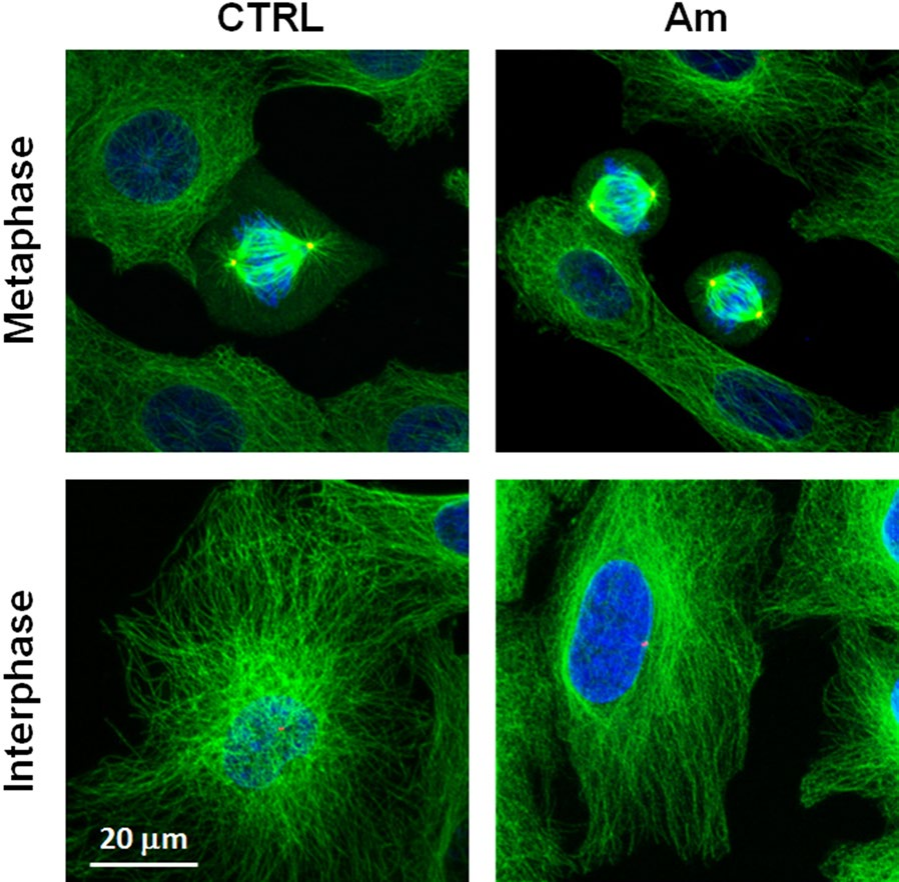
Effect of amitozyn on the microtubules of human A549 cells. A549 cells were treated with 0 or 30 μg/mL amitozyn for 24 h. Cells were fixed with 2 % paraformaldehyde, stained with anti-β-tubulin (*green*), anti-γ-tubulin (*red*) and DAPI (*blue*) and analysed by confocal microscope

To analyse the effect of amitozyn on the microtubules of *P. falciparum,* amitozyn treated and untreated (control) RBC infected with *P. falciparum* (3G8 strain) were stained with anti-α-tubuline antibodies. The treatment of *P. falciparum* with 30 μg/mL amitozyn destroyed the filamentous structure of parasite microtubules at the ring and trophozoite stages (Fig. 2a, b). Importantly, the same amitozyn concentration (30 μg/mL) did not induce depolymerization of the metaphase and interphase microtubules in human A549 cells and did not lead to centrosome multiplication (Fig. 3). Obtained results demonstrate that amitozyn disrupts the microtubule organization in *P. falciparum* substantially more effectively than in mammalian cells. It is likely that microtubule depolymerization can be one of the major causes of its antiparasitic effect.

### Amitozyn halts *Plasmodium falciparum* development at trophozoite stage

**Fig. 4:**
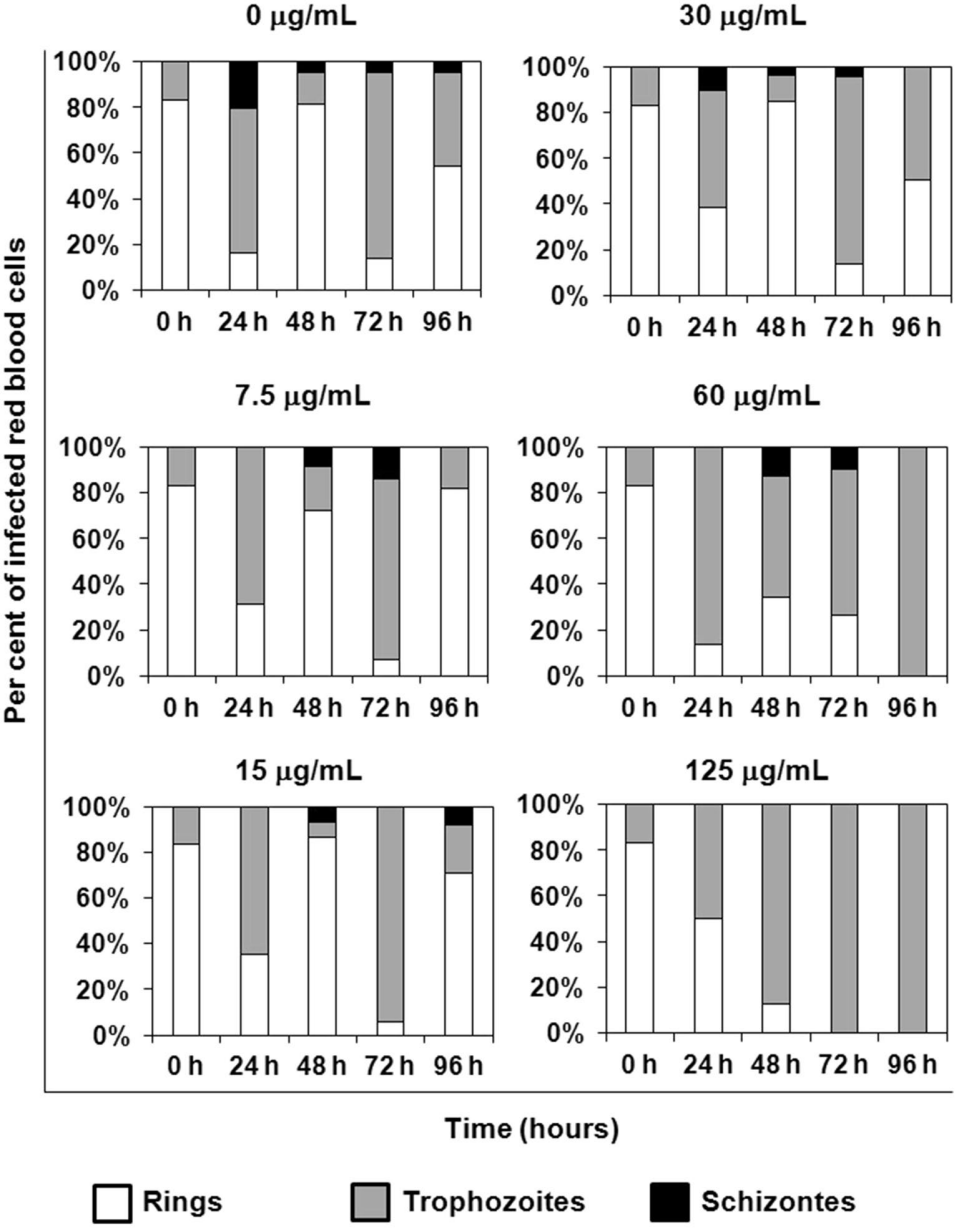
Effect of amitozyn on the parasite developmental stage distribution. *Plasmodium falciparum* (3G8) culture was synchronized at the ring stage using 5 % d-sorbitol. iRBC were treated with 0–125 μg/mL amitozyn for 24, 48, 72, and 96 h and then stained by Giemsa’s method. The relative ratio of rings, trophozoites and schizonts was analysed by inverted light microscope as described in “Methods”

To analyse whether amitozyn treatment affects a specific stage of parasite development, the changes in the ratio of rings, trophozoites and schizonts in the culture of *P. falciparum* (3G8) synchronized at the ring stage were measured. Treatment of synchronized *P. falciparum* with 0–30 μg/mL amitozyn did not change the profile of normal stage distribution of parasites (Fig. 4). However, after treatment with higher (60–125 μg/mL) amitozyn concentrations accumulation of trophozoites was clearly observed at time points of 48, 72 and 96 h (Fig. 4).

These results suggest that treatment of *P. falciparum* with high amitozyn concentrations halts the parasite cell cycle and leads to accumulation of the trophozoite stage.

### Amitozyn is not cytotoxic for human red blood cells

To estimate the toxic effects of amitozyn on human RBC, freshly prepared RBC (O^+^) were treated with amitozyn (0–500 μg/mL) for 24, 48, 72, and 96 h and analysed for the concentration of released LDH. RBC treated with complete RPMI media supplemented with 0.1 % Triton X-100 were used as a positive control.

**Fig. 5:**
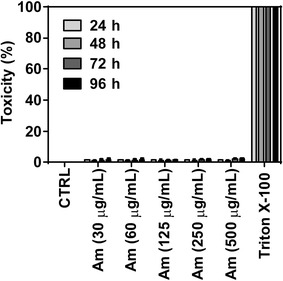
Cytotoxic effect of amitozyn on human erythrocytes. Human erythrocytes (O^+^) at 2 % haematocrit were treated with 0, 30, 60, 125, 250, and 500 μg/mL amitozyn or complete RPMI supplemented with 0.1 % Triton X-100 (positive control) for 24, 48, 72, and 96 h. Cytotoxity was determined by LDH kit as described in “Methods”

Obtained results show that treatment of RBC with high doses of amitozyn (30–500 μg/mL) for 96 h did not increase the level of released LDH (Fig. 5), showing its low toxicity for RBC.

These results demonstrate that the semi-synthetic drug amitozyn is not toxic for human RBC and does not provoke haemolysis.

### Amitozyn enhances the antiparasitic effects of chloroquine, pyrimethamine and artemisinin

To estimate the combinatory effects of amitozyn with CQ, pyrimethamine and ART isobolographic analyses were performed.

**Fig. 6:**
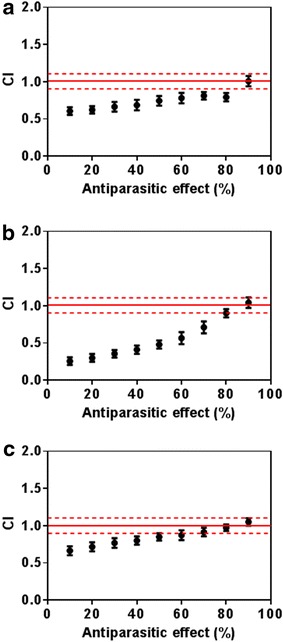
Isobolographic analysis of combined effects of amitozyn with chloroquine, pyrimethamine and artemisinin in the culture of *Plasmodium falciparum* (3G8). Calculation of CI values of **a** amitozyn/chloroquine, **b** amitozyn/pyrimethamine and **c** amitozyn/artemisinin combinations at antiparasitic effects from 10 to 90 %. Human RBC at 2 % haematocrit were infected with 3G8 line of *P. falciparum.* At 0.5 % parasitaemia iRBC were treated with amitozyn (0–60 μg/mL), chloroquine (0–30 nM), pyrimethamine (0–20 nM), artemisinin (0–4 nM), and the combinations of amitozyn with chloroquine, pyrimethamine and artemisinin in the molar ratio 778:1, 778:1 and 11,679:1, respectively, for 96 h. CI was calculated as described in “Methods”

Synergistic effect of amitozyn/chloroquine was observed at APE of 10–80 % and a nearly additive effect was observed at APE of 90 % (Fig. 6a; Additional files 2, 3). Combination of amitozyn with pyrimethamine provoked a synergistic anti-malarial effect at APE of 10–70 % and a nearly additive effect at APE of 90 % (Fig. 6b; Additional files 2, 3) Combination of amitozyn with ART provoked a synergistic anti-malarial effect at APE of 10–40 % and a nearly additive effect at APE of 50–90 % (Fig. 6c; Additional files 2, 3). Furthermore, the dose reduction index (DRI) for each drug in their combination was determined. Combination of amitozyn with CQ in the molar ratio 778:1 reduces the effective dose of amitozyn from about three to eight times depending on the APE (Additional file 4). In contrast, the combination of CQ with amitozyn in the molar ratio 1:778 decreased its effective dose about two times for APE of 10–90 % (Additional file 4). Combination of amitozyn with pyrimethamine in the molar ratio 778:1 reduced the amitozyn dose three to four times, however, the combination of pyrimethamine with amitozyn in the molar ratio 1:778 reduced the pyrimethamine dose from two to 44 times depending on the level of APE (Additional file 5). More modest DRI was observed for the combination of amitozyn with ART. The combination of amitozyn with ART in the molar ratio 11,679:1 reduced the effective dose of amitozyn two to three times and the combination of ART with amitozyn in the molar ratio 1:11,679 reduced the effective dose of ART four times (Additional file 6). The combinatory effect of amitozyn with pyrimethamine has a more strong synergistic effect compared to the combinatory effect of amitozyn with CQ or ART.

The antiproliferative combinatory effects of amitozyn paired with CQ, pyrimethamine and ART on the human endothelial cells were also tested (HUVEC, Additional file 7). The combination of amitozyn with CQ or pyrimethamine at a molar ratio 1:1 has an additive antiproliferative effect on the HUVEC at all levels of growth inhibition. The combination of amitozyn with ART at a molar ratio 1:1 has an additive antiproliferative effect at growth inhibition levels of 10–70 % and slightly antagonistic antiproliferative effect at growth inhibition levels of 80–90 % (Additional file 8).

These results suggest that combinations of amitozyn/chloroquine, amitozyn/pyrimethamine and amitozyn/artemisinin cause synergistic and nearly additive anti-malarial effects in the culture of *P. falciparum* and an additive or slightly antagonistic, antiproliferative effect on the human cells.

## Discussion

For the first time, the anti-malarial effect of the semi-synthetic anticancer drug amitozyn has been described. It was shown earlier that this drug demonstrates anticancer activity in different models and acts by the modulation of microtubule polymerization [15, 21]. The main mechanism of amitozyn action in mammalian cells is the perturbation of the mitotic spindle, which leads to mitotic checkpoint activation, mitotic arrest and apoptosis [15]. Similar antimicrotubule effects of amitozyn were observed in the *P. falciparum* parasite. The microtubules of *P. falciparum* contribute to its shape and integrity, and may be involved in motility and invasion of the parasite [22, 23]. Moreover, spindle microtubules are re-organized into structures that appear to facilitate the partition of daughter cells and their organelles during parasite development in erythrocytes [24]. It was demonstrated that amitozyn depolymerizes the microtubules of the *Plasmodium* parasite at intra-erythrocytic stages (Fig. 2) and leads to post-ring stage accumulation (Fig. 4). Importantly, the antimicrotubule effect of amitozyn in the culture of *P. falciparum* is evident at substantially lower concentrations than in the culture of mammalian cells. For example, amitozyn depolymerizes the parasite microtubules at 30 μg/mL but does not induce any visible effect at the same concentration in the culture of the lung adenocarcinoma A549 (Fig. 3). It is likely that the sensitivity of parasite microtubules to amitozyn is a result of different affinities of this drug to human and parasitic tubulin due the minor sequence differences between tubulins [25–28]. A stronger sensitivity of tubulin depolymerization may result in a more potent anti-malarial effect. Obtained results demonstrate the inhibitory coefficient 50 % (IC_50_) for the CS2, 3G8 and NF54 of *P. falciparum* lines is significantly lower than for the mammalian cell lines (Fig. 1b). Relative resistance of mammalian cells to amitozyn varied from three (HeLa cells) up to 15 times (HUVEC) compared to *P. falciparum* (Fig. 1b). This is a promising drug feature which potentially may allow the application of amitozyn for malaria treatment with minimal secondary effects to humans. The non-toxicity of amitozyn for RBC, even at extremely high concentrations (500 μg/mL), represents a supplemental advantageous point of this drug (Fig. 5).

Drug combinations are extensively used in medical practice to enhance their anti-malarial impact and simultaneously decrease their doses. Synergistic or nearly additive drug interactions attenuate the secondary effects and significantly improve the antiparasite treatment. Multiple data describing interactions of the anti-malarial drugs in vitro were reported [29–36].

IC_50_ levels of amitozyn (7.7 μM), CQ (29.4 nM), pyrimethamine (6.8 nM), and ART (1.5 nM) were significantly diminished when these drugs were used in combinations. The combination of amitozyn with CQ resulted in a synergistic interaction at a range of APE from 10 up to 80 % and a nearly additive effect at APE of 90 % (Fig. 6a; Additional files 2, 3). Amitozyn and CQ target different physiological mechanisms of parasites which are important for its viability. The anti-malarial effect of CQ results from its accumulation at high levels in the acidic lysosomal food vacuole of the parasite and its binding to haematin. The food vacuole is the site of haemoglobin degradation in the parasite, and iron (II) haem is released as a by-product. Under normal circumstances, the iron (II) haem is oxidized to iron (III) haematin and sequestered into a polymer of β-haematin as an inert pigment called hemozoin. CQ binds to haematin and disrupts its polymerization. It has been demonstrated that free haematin or CQ-haematin complexes are membrane interactive and toxic to the parasite [37]. Inhibition of haematin polymerization together with microtubule disruption during intracellular development of the parasite may lead to a synergistic anti-malarial effect. Interestingly, the synergistic impact of amitozyn/chloroquine combinations was inversely proportional to the growth inhibition level. The synergistic interaction was observed at APE of 10 and 20 %, a moderate synergistic effect was observed at APE of 30–60 %, a slight synergistic effect was observed at APE of 70–80 %, and a nearly additive effect was observed at APE of 90 % (Fig. 6a; Additional files 2, 3).

Combinations of amitozyn with pyrimethamine induced a pronounced synergistic effect at APE of 10–60 %, a moderate synergistic effect at APE of 70 % and a nearly additive effect at APE of 80 and 90 % (Fig. 6b; Additional files 2, 3). As known, pyrimethamine inhibits dihydrofolate reductase (DHFR) from the parasite to a greater degree than DHFR from the host and thus shows a selective toxicity towards the parasite [38]. DHFR is a ubiquitous enzyme that participates in the recycling of folates by reducing dihydrofolate to tetrahydrofolate [39]. Inhibition of DHFR prevents the formation of fully reduced tetrahydrofolate, which participates in purine, pyrimidine and amino acid biosynthetic pathways [40]. Lower levels of tetrahydrofolate decrease the conversion of glycine to serine, reduce methionine synthesis and lower thymidylate levels with a subsequent arrest of DNA replication [41–43]. The toxic effect of pyrimethamine on the parasite reaches a peak in the late erythrocytic schizont stage, precisely when DNA synthesis peaks [44]. Simultaneous affection of DNA replication and microtubule integrity may significantly inhibit the parasite production and lead to the synergistic anti-malarial effect.

The combinations of amitozyn with ART slightly enhanced the antiparasitic effect of both drugs. A moderate synergistic interaction was observed at APE of 10–40 % and a nearly additive effect at APE of 50–90 % (Fig. 6c; Additional files 2, 3). The precise mechanism of ART action is not completely clear. It was reported that ART specifically inhibits the PfATP6 enzyme, which is essential for oxidative metabolism in the parasite [45], but this mechanism was questioned later [46]. Another possible mechanism of ART action is the production of carbon-centred free radicals [47] or production of reactive oxygen species within the mitochondria of the malarial parasite [48]. Furthermore, a number of studies have shown that ART covalently reacts with several parasitic proteins [49]. Combinations of amitozyn with ART moderately enhanced the anti-malarial effect in contrast to amitozyn/chloroquine and amitozyn/pyrimethamine combinations. Importantly, amitozyn combined with CQ, pyrimethamine and ART in the culture of HUVEC did not induce the synergistic antiproliferative effect, in contrast to the culture of *P. falciparum.*

## Conclusions

The anticancer drug amitozyn effectively inhibits *P. falciparum* proliferation in doses significantly lower than those inhibiting human cell growth. Moreover, this drug is not toxic for RBC and enhances the anti-malarial effect of CQ, pyrimethamine and ART in the in vitro culture of *P. falciparum,* but does not enhance the cytostatic effect of mentioned drugs in the culture of human cells. All these results provide a strong basis for future detailed investigations of the anti-malarial effect of amitozyn.

It has been shown for the first time that the semi-synthetic drug amitozyn has anti-malarial properties and might be a useful candidate for treatment of malaria, induced by *P. falciparum,* alone or in combinations with other anti-malarial drugs.

## Additional files

10.1186/s12936-015-0952-4 Antiparasitic effect of amitozyn pretreatment. Human RBC at 2 % haematocrit were infected with 3G8 strain of *P. falciparum*. At 1 % of parasitaemia iRBC were pretreated with 0, 15 and 30 μg/mL amitozyn for 24 or 48 h. After wash (three times) iRBC were diluted to parasitaemia 0.5 % and released in the drug-free medium for 144 h. At time points 48, 96 and 144 h iRBC were collected and stained by Giemsa’s method. Parasitaemia was analysed by inverted light microscope. The data were statistically treated and plotted on the graphs using GraphPad software. Error bars present the standard deviation.
10.1186/s12936-015-0952-4 Antiparasitic effect of (A) amitozyn/chloroquine combinations in the ratio 778:1, (B) chloroquine/amitozyn combinations in the ratio 1:778, (C) amitozyn/pyrimethamine combinations in the ratio 778:1, (D) pyrimethamine/amitozyn combinations in the ratio 1:778, (E) amitozyn/artemisinin combinations in the ratio 11,679:1, (F) artemisinin/amitozyn combinations in the ratio 1:11,679.
10.1186/s12936-015-0952-4 CI values of amitozyn/chloroquine, amitozyn/pyrimethamine and amitozyn/artemisinin combinations at different levels of antiparasitic effect.
10.1186/s12936-015-0952-4 Drug reduction index of amitozyn/chloroquine and chloroquine/amitozyn combinations.
10.1186/s12936-015-0952-4 Drug reduction index of amitozyn/pyrimethamine and pyrimethamine/amitozyn combinations.
10.1186/s12936-015-0952-4 Drug reduction index of amitozyn/artemisinin and artemisinin/amitozyn combinations.
10.1186/s12936-015-0952-4 Antiproliferative effect of amitozyn combinations with chloroquine, pyrimethamine and artemisinin on the HUVEC. (A) amitozyn/chloroquine (B) chloroquine/amitozyn (C) amitozyn/pyrimethamine (D) pyrimethamine/amitozyn (E) amitozyn/artemisinin (F) artemisinin/amitozyn combinations. HUVEC were treated with amitozyn (0-500 μg/mL), chloroquine (0-200 μM), pyrimethamine (0-160 μM), artemisinin (0-160 μM), and the combinations of amitozyn with chloroquine, pyrimethamine and artemisinin in the molar ratio 1:1 for 72 h. Then cells were fixed with 3.7 % formaldehyde and stained with 0.1 % crystal violet. Per cent of growth inhibition was analysed as described in “Methods”.
10.1186/s12936-015-0952-4 Isobolographic analysis of combined effects of amitozyn with chloroquine, pyrimethamine and artemisinin on the HUVEC.

## References

[1] Mueller I, Zimmerman PA, Reeder JC (2007). *Plasmodium malariae* and *Plasmodium ovale*—the “bashful” malaria parasites. Trends Parasitol.

[2] Collins WE (2012). *Plasmodium knowlesi*: a malaria parasite of monkeys and humans”. Annu Rev Entomol.

[3] Nadjm B, Behrens RH (2012). Malaria: an update for physicians. Infect Dis Clin North Am.

[4] Sarkar PK, Ahluwalia G, Vijayan VK, Talwar A (2009). Critical care aspects of malaria. J Intensive Care Med.

[5] Baird JK (2013). Evidence and implications of mortality associated with acute *Plasmodium vivax* malaria. Clin Microbiol Rev.

[6] Aguiar AC, Rocha EM, Souza NB, França TC, Krettli AU (2012). New approaches in antimalarial drug discovery and development. Mem Inst Oswaldo Cruz.

[7] Dondorp AM, Nosten F, Yi P, Das D, Phyo AP, Tarning J (2009). Artemisinin resistance in *Plasmodium falciparum* malaria. N Engl J Med.

[8] WHO (2010). Guidelines for the treatment of malaria.

[9] WHO (2010). Global report on antimalarial drug efficacy and drug resistance: 2000–2010.

[10] Wildbolz A (1973). Methotrexate in the therapy of malaria. Ther Umsch.

[11] Pouvelle B, Farley PJ, Long CA, Taraschi TF (1994). Taxol arrests the development of blood-stage *Plasmodium falciparum* in vitro and *Plasmodium* chabaudi adami in malaria-infected mice. J Clin Invest.

[12] Usanga EA (1986). Mitotic inhibitors arrest the growth of *Plasmodium falciparum*. FEBS Lett.

[13] Nair L, Bhasin VK (1994). Cure with cisplatin (II) or murine malaria infection and in vitro inhibition of a chloroquine-resistant *Plasmodium falciparum* isolate. Jpn J Med Sci Biol.

[14] Kreidenweiss A (2008). Comprehensive study of proteasome inhibitors against *Plasmodium falciparum* laboratory strains and field isolates from Gabon. Malar J.

[15] Hermant B, Gudrun A, Potopalsky AI, Chroboczek J, Tcherniuk SO (2013). Amitozyn impairs chromosome segregation and induces apoptosis via mitotic checkpoint activation. PLoS One.

[16] Moll K, Ljungstrom I, Perlmann H, Scherf A, Mats Wahlgren M. Methods in Malaria Research, 5th edn. Virginia: MR4/ATCC; 2008.

[17] Shapiro HM, Mandy F (2007). Cytometry in malaria: moving beyond Giemsa. Cytom A..

[18] Chou TC (2006). Theoretical basis, experimental design, and computerized simulation of synergism and antagonism in drug combination studies. Pharmacol Rev.

[19] Chou TC, Chou TC, Rideout DC (1991). The median-effect principle and the combination index for quantitation of synergism and antagonism. Synergism and antagonism in chemotherapy.

[20] Tonkin CJ, van Dooren GG, Spurck TP, Struck NS, Good RT, Handman E (2004). Localization of organellar proteins in *Plasmodium falciparum* using a novel set of transfection vectors and a new immunofluorescence fixation method. Mol Biochem Parasitol.

[21] Fil’chenkov OO, Zavelevych MP, Khranovs’ka NM, Zaïka LA, Potopal’s’ky AI. Modified alkaloids from *Chelidonium majus* L. induce G2/M arrest, caspase-3 activation, and apoptosis in human acute lymphoblastic leukemia MT-4 cells. Ukr Biokhim Zh 2006;78:81–7. 17290785

[22] Bell A (1998). Microtubule inhibitors as potential antimalarial agents. Parasitol Today.

[23] Bejon PA, Bannister LH, Fowler RE, Fookes RE, Webb SE, Wright A (1997). A role for microtubules in *Plasmodium falciparum* merozoite invasion. Parasitology.

[24] Gerald N, Mahajan B, Kumar S (2011). Mitosis in the human malaria parasite *Plasmodium falciparum*. Eukaryot Cell.

[25] Delves CJ, Ridley RG, Goman M, Holloway SP, Hyde JE, Scaife JG (1989). Cloning of a beta-tubulin gene from *Plasmodium falciparum*. Mol Microbiol.

[26] Holloway SP, Sims PF, Delves CJ, Scaife JG, Hyde JE (1989). Isolation of alpha-tubulin genes from the human malaria parasite, *Plasmodium falciparum*: sequence analysis of alpha-tubulin. Mol Microbiol.

[27] Holloway SP, Gerousis M, Delves CJ, Sims PF, Scaife JG, Hyde JE (1990). The tubulin genes of the human malaria parasite *Plasmodium falciparum*, their chromosomal location and sequence analysis of the alpha-tubulin II gene. Mol Biochem Parasitol.

[28] Wesseling JG, Dirks R, Smits MA, Schoenmakers JG (1989). Nucleotide sequence and expression of a beta-tubulin gene from *Plasmodium falciparum*, a malarial parasite of man. Gene.

[29] Winstanley PA, Mberu EK, Szwandt IS, Breckenridge AM, Watkins WM (1995). In vitro activities of novel antifolate drug combinations against *Plasmodium falciparum* and human granulocyte CFUs. Antimicrob Agents Chemother.

[30] Ohrt C, Willingmyre GD, Lee P, Knirsch C, Milhous W (2002). Assessment of azithromycin in combination with other antimalarial drugs against *Plasmodium falciparum* in vitro. Antimicrob Agents Chemother.

[31] Canfield CJ, Pudney M, Gutteridge WE (1995). Interactions of atovaquone with other antimalarial drugs against *Plasmodium falciparum* in vitro. Exp Parasitol.

[32] Co EM, Dennull RA, Reinbold DD, Waters NC, Johnson JD (2009). Assessment of malaria in vitro drug combination screening and mixed-strain infections using the malaria Sybr green I-based fluorescence assay. Antimicrob Agents Chemother.

[33] Agarwal D, Sharma M, Dixit SK, Dutta RK, Singh AK, Gupta RD (2015). In vitro synergistic effect of fluoroquinolone analogues in combination with artemisinin against *Plasmodium falciparum*; their antiplasmodial action in rodent malaria model. Malar J.

[34] He Z, Chen L, You J, Qin L, Chen X (2009). In vitro interactions between antiretroviral protease inhibitors and artemisinin endoperoxides against *Plasmodium falciparum*. Int J Antimicrob Agents.

[35] Bhattacharya A, Mishra LC, Bhasin VK (2008). In vitro activity of artemisinin in combination with clotrimazole or heat-treated amphotericin B against *Plasmodium falciparum*. Am J Trop Med Hyg.

[36] Nakornchai S, Konthiang P (2006). Activity of azithromycin or erythromycin in combination with antimalarial drugs against multidrug-resistant *Plasmodium falciparum* in vitro. Acta Trop.

[37] Hempelmann E (2007). Hemozoin biocrystallization in *Plasmodium falciparum* and the antimalarial activity of crystallization inhibitors. Parasitol Res.

[38] Ferone R, Burchall JJ, Hitchings GH (1969). *Plasmodium berghei* dihydrofolate reductase. Isolation, properties, and inhibition by antifolates. Mol Pharmacol.

[39] Schnell JR, Dyson HJ (2004). Wright PE Structure, dynamics, and catalytic function of dihydrofolate reductase. Annu Rev Biophys Biomol Struct.

[40] Ferone R (1977). Folate metabolism in malaria. Bull World Health Organ.

[41] Schellenberg KA, Coatney GR (1961). The influence of antimalarial drugs on nucleic acid synthesis in *Plasmodium gallinaceum* and *Plasmodium berghei*. Biochem Pharmacol.

[42] Gutteridge WE, Trigg PI (1971). Action of pyrimethamine and related drugs against *Plasmodium knowlesi* in vitro. Parasitology.

[43] Newbold CI, Boyle DB, Smith CC, Brown KN (1982). Stage specific protein and nucleic acid synthesis during the asexual cycle of the rodent malaria *Plasmodium chabaudi*. Mol Biochem Parasitol.

[44] Hyde JE (1990). The dihydrofolate reductase–thymidylate synthetase gene in the drug resistance of malaria parasites. Pharmacol Ther.

[45] Eckstein-Ludwig U, Webb RJ, Van Goethem ID, East JM, Lee AG, Kimura M (2003). Artemisinins target the SERCA of *Plasmodium falciparum*. Nature.

[46] Arnou B, Montigny C, Morth JP, Nissen P, Jaxel C, Møller JV (2011). The *Plasmodium falciparum* Ca(2+)-ATPase PfATP6: insensitive to artemisinin, but a potential drug target. Biochem Soc Trans.

[47] Meshnick SR (2002). Artemisinin: mechanisms of action, resistance and toxicity. Int J Parasitol.

[48] Li W, Mo W, Shen D, Sun L, Wang J, Lu S (2005). Yeast model uncovers dual roles of mitochondria in action of artemisinin. PLoS Genet.

[49] Meshnick SR, Little B, Yang YZ (1994). Alkylation of proteins by artemisinin. Biochem Pharm.

