# Supplementary figures and images for: Anti-malarial effect of semi-synthetic drug amitozyn
Source: Malar J. 2015 Oct 29;14:425. doi: 10.1186/s12936-015-0952-4 (PMC4625481; doi:10.1186/s12936-015-0952-4)

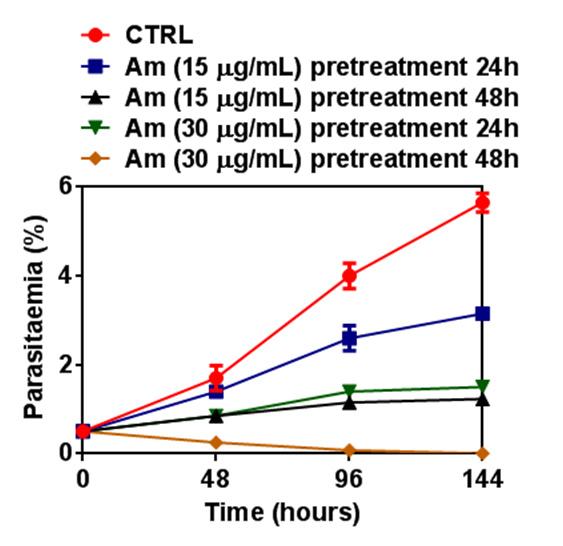

Supplement: Supplementary file 1 — 10.1186/s12936-015-0952-4 Antiparasitic effect of amitozyn pretreatment. Human RBC at 2 % haematocrit were infected with 3G8 strain of P. falciparum. At 1 % of parasitaemia iRBC were pretreated with 0, 15 and 30 μg/mL amitozyn for 24 or 48 h. After wash (three times) iRBC were diluted to parasitaemia 0.5 % and released in the drug-free medium for 144 h. At time points 48, 96 and 144 h iRBC were collected and stained by Giemsa’s method. Parasitaemia was analysed by inverted light microscope. The data were statistically treated and plotted on the graphs using GraphPad software. Error bars present the standard deviation. [file 12936_2015_952_MOESM1_ESM.jpg]

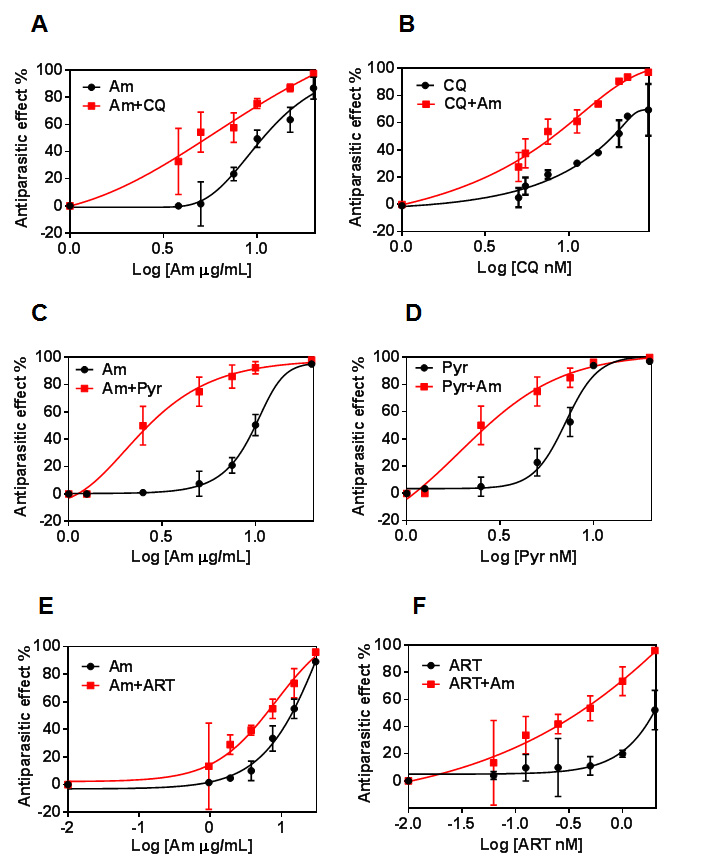

Supplement: Supplementary file 2 — 10.1186/s12936-015-0952-4 Antiparasitic effect of (A) amitozyn/chloroquine combinations in the ratio 778:1, (B) chloroquine/amitozyn combinations in the ratio 1:778, (C) amitozyn/pyrimethamine combinations in the ratio 778:1, (D) pyrimethamine/amitozyn combinations in the ratio 1:778, (E) amitozyn/artemisinin combinations in the ratio 11,679:1, (F) artemisinin/amitozyn combinations in the ratio 1:11,679. [file 12936_2015_952_MOESM2_ESM.jpg]

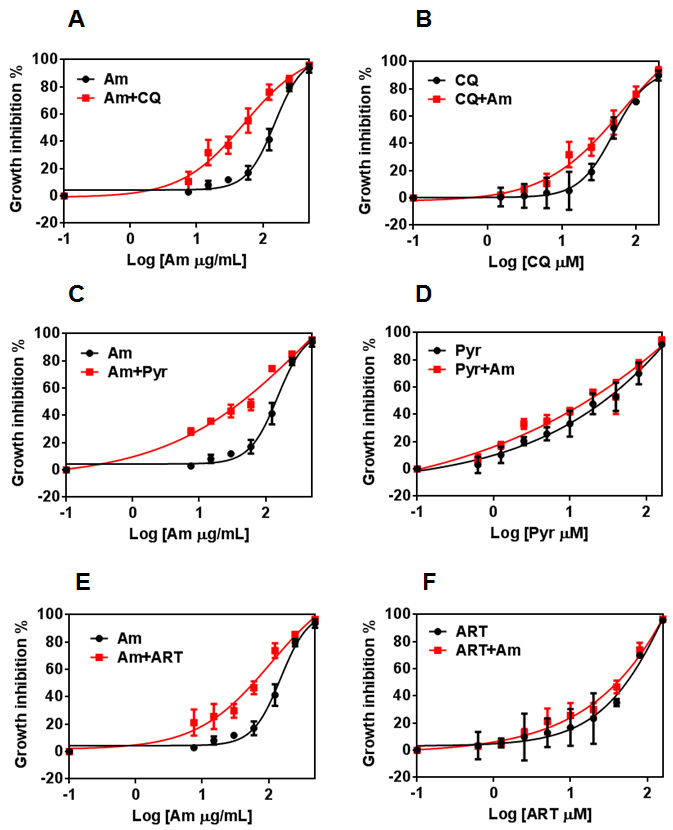

Supplement: Supplementary file 7 — 10.1186/s12936-015-0952-4 Antiproliferative effect of amitozyn combinations with chloroquine, pyrimethamine and artemisinin on the HUVEC. (A) amitozyn/chloroquine (B) chloroquine/amitozyn (C) amitozyn/pyrimethamine (D) pyrimethamine/amitozyn (E) amitozyn/artemisinin (F) artemisinin/amitozyn combinations. HUVEC were treated with amitozyn (0-500 μg/mL), chloroquine (0-200 μM), pyrimethamine (0-160 μM), artemisinin (0-160 μM), and the combinations of amitozyn with chloroquine, pyrimethamine and artemisinin in the molar ratio 1:1 for 72 h. Then cells were fixed with 3.7 % formaldehyde and stained with 0.1 % crystal violet. Per cent of growth inhibition was analysed as described in “Methods”. [file 12936_2015_952_MOESM7_ESM.jpg]

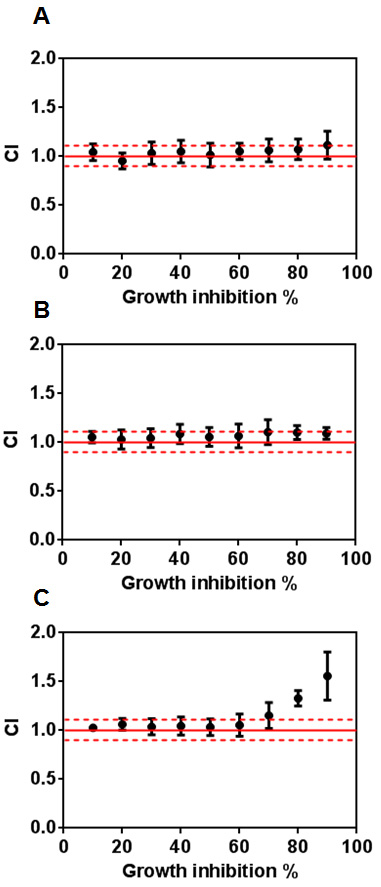

Supplement: Supplementary file 8 — 10.1186/s12936-015-0952-4 Isobolographic analysis of combined effects of amitozyn with chloroquine, pyrimethamine and artemisinin on the HUVEC. [file 12936_2015_952_MOESM8_ESM.jpg]
